# POISED-5, a portable on-board electrochemical impedance spectroscopy biomarker analysis device

**DOI:** 10.1007/s10544-019-0406-9

**Published:** 2019-07-04

**Authors:** M. Anne Sawhney, R. S. Conlan

**Affiliations:** 1 Swansea University Medical School, Singleton Park, Swansea, SA2 8PP UK; 2 Centre for NanoHealth, Swansea University, Singleton Park, Swansea, SA2 8PP UK

**Keywords:** EIS, Point-of-care, POISED-5

## Abstract

Point-of-care medical devices offer the potential for rapid biomarker detection and reporting of medical conditions, thereby bypassing the requirements for offline clinical laboratory facilities in many cases. Label-free electrochemical techniques are suitable for use in handheld diagnostic devices due the inherent electronic detection modality and low requirement for processing reagents. While electrochemical impedance sensing is widely used in tissue analysis such as body composition measurement, its use in point-of-care patient testing is yet to be widely adopted. Here we have considered a number of issues currently limiting the translation of electrochemical impedance sensing into clinical biosensor devices. Specifically, we have addressed the current requirement for these sensors to be connected to an external processor by applying a minimum number of frequencies required for optimized biomarker detection, and subsequently delivering analytics within the measurement device. The POISED-5 device was evaluated using a sensor for the ovarian cancer biomarker cancer antigen 125 (CA125), demonstrating performance comparable to standard laboratory equipment, with direct interpretation of response signal amplitude substituting traditional impedance component calculation and model fitting.

## Introduction

Point-of-care (POC) biosensing devices for rapid diagnosis outside a clinical laboratory environment are expected to feature widely in future patient care (Jung et al., 2015; Weigl et al. 2014). Among several streams of development, electrochemical sensing technologies have gained attention due to their label-free capability and relatively few procedural steps required to obtain measurements (Veloso et al. 2012). While amperometric sensing features frequently in such devices, this approach can be restrictive as it applies voltages that can affect the integrity or binding of biological material (Xu and Davis 2014). As an alternative, electrochemical impedance spectroscopy (EIS) allows detection of subtle electrochemical changes with minimal complexity, has a low energy demand and is tunable through parameter optimization (Kokkinos et al. 2016).

Impedance sensing is already employed in clinical diagnosis such as in electrical impedance myography (Sanchez and Rutkove 2017), blood impedance analysis (Ghafar-Zadeh et al. 2010) and electrical impedance tomography (Adler and Boyle 2017). Measuring impedances over more than one frequency is considered an advantage for certain applications, with commercially-available devices for microbiology (Ramirez et al. 2009) and cervical screening (Leeson 2014). These bioimpedance methods apply electrical signals directly to patient tissue, or to patient samples in a laboratory, both of which require specialized medical equipment and training (Shaw 2016).

As an electrochemical analysis technique, EIS has historically been used industrially for monitoring corrosion (Angelini et al. 2006) and battery efficiency (Din et al. 2016), while its use for medical applications is less well established (Pathiraja et al. 2017). EIS relies on the physical and chemical properties of molecules to alter the transmission of electrical current (Carminati and Li 2017). A larger density of obstructions will have a greater effect on current with a proportional scale, though the quantity of this effect is dependent on several variables. By standardizing conditions, the addition of current-obstructing molecules (such as protein biomarkers) will alter a system’s internal impedance by a defined quantity (Chang and Park 2010).

**Fig. 1:**
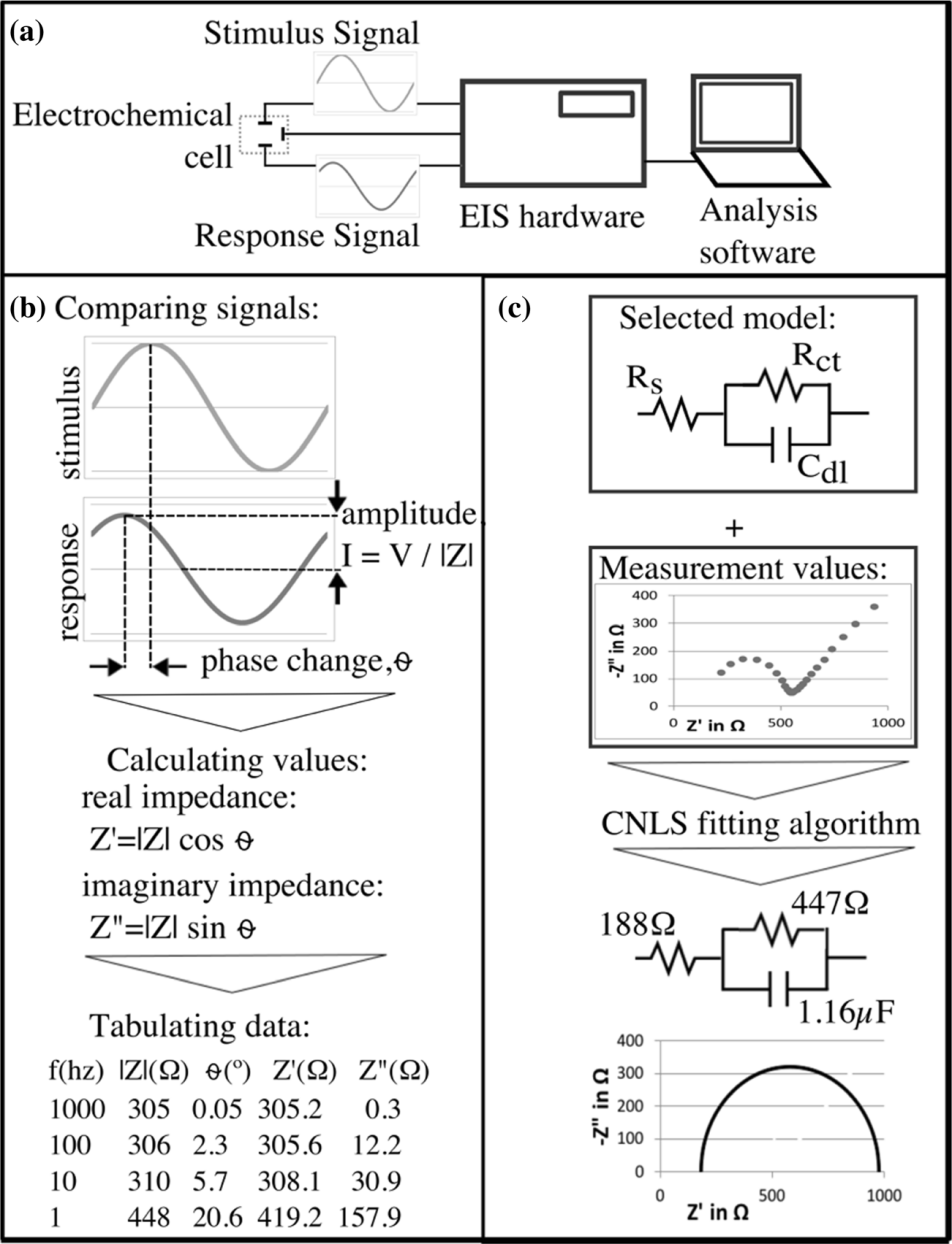
Generic EIS processes. **a** Hardware generates stimulus signals, conditions response signals and sends digital data to analysis software (**b**) Basic impedance analysis extracts amplitude and phase change, as well as calculating real and imaginary components for each frequency applied (**c**) Further analysis of impedance data selects an equivalent circuit model expected to simulate the electrochemical phenomena of the system, then a mathematical algorithm is used to determine the best fit of model components to the actual measurement data

EIS can be performed using a variety of bench-top hardware, including electrochemical suites with a frequency response analyzer (FRA), dedicated impedance analyzers or basic inductance-capacitance-resistance meters. For potentiometric measurements, a voltage (V) signal is generated and the response is detected in the form of an electrical current (I); the reverse is true for galvanostatic systems. In either case, to achieve an EIS measurement, a stimulus signal must be generated across the sample under investigation, in the pattern of a sine wave (Fig. 1a). This allows a quantitative comparison of the stimulus with the resulting response in two respects, amplitude and phase. An amplitude reduction in the response signal is expected, due to energy lost by the signal when passing through the sample under test and relates inversely to the impedance magnitude (|Z|) (Fig. 1b). In all systems, this contributes to electrical resistance or real impedance (Z’). A phase change (⍬) would occur as a result of capacitive or inductive properties of the sample, which produces imaginary impedance (Z”).

Measurements by EIS equipment are usually performed over a range of frequencies, giving numerical raw data. Interpreting the overall significance of these magnitude and phase changes is often achieved by calculating real and imaginary components, and in some cases, fitting actual values to an idealized model. Conventionally, models are in the form of equivalent circuits, to which software compares measurement data using one of several complex nonlinear least-squares (CNLS) minimization methods (Fig. 1c). For example, the ubiquitous R(RC) model equates solution resistance to a series resistor (R_s_), the interfacial double-layer capacitance as a capacitor (C_dl_) and the interfacial charge-transfer resistance as a resistor (R_ct_).

This traditional approach allows various frequency ranges, input amplitudes and models to be tested for a given system as part of investigation into molecular-level electrochemical processes. Statistical tools can aid in determining the success of selected parameters, including stability testing with Kramer-Kronigs relations (Park et al. 2006) and calculation of fitting error to indicate appropriateness of a given model.

A diverse set of electrochemical materials may be employed for impedance detection, provided consistency of background impedance can be assured both before and after exposure to the test sample (Millner et al. 2012). Since impedance is not specific to a material, EIS is generally used in combination with other physical and chemical characterization methods to determine the specific sources of impedance changes in a system. Such analysis may include scanning electron microscopy to assess surface roughness (Petroni et al. 2017), energy-dispersive X-ray spectroscopy for elemental analysis (Bhide et al. 2018) or surface plasmon resonance to confirm material adsorption (Jolly et al. 2015). Once established, method parameters including circuit model are fixed (Orazem and Tribollet 2007), and frequency ranges used for EIS measurements can be shortened a minimum requirement (Niloy et al. 2017; Ramaswamy et al. 2013; Tsai and Wang 2016; Grassini et al. 2015). For example, a change of resistance in contaminated milk could be detected by measuring with only one frequency, while resistance remained the same over the rest of the tested frequency range (Durante et al. 2016). Once the frequencies of interest have been established, additional frequency measurements serve only to extend the analysis time while providing no additional relevant information (Seoane et al. 2012).

Another major consideration is model fitting of EIS data to generate key values, such as charge transfer resistance. Conventional analysis requires hundreds of post-measurement calculations as part of a CNLS algorithm to generate equivalent circuit values. Specialized EIS software include features such as selection of a best-fit model from several equivalent circuit models, plotting data graphically and calculating percentage error for each component value, which, due to memory and processing requirements, involve a separate computing platform.

Portable EIS devices containing many of the same features as lab-bench suites (Pruna et al. 2018) are available, however, model fitting algorithms are proprietary and cannot therefore be adapted, and require training in electrochemical theory, including interpretation of equivalent circuit models and/or complex graphical data making them impractical for use in POC environments. Taken together, the limitations of currently available EIS analysis platforms favor the development of customizable EIS devices that meet the needs of the end user and the parameters of a particular measurement.

Portable EIS sensor development is often application dependent (Table 1). Analysis of non-faradaic system measurements, such as corrosion of industrial coatings, spans low frequency ranges (Hoja and Lentka 2013), while bioimpedance measurements often span from kilohertz (kHz) to sub-hertz bandwidths, the latter requiring large capacitances considered impractical to implement in printed electronics (Mahnashi and Alzaher 2012). This milli-hertz region is increasingly critical to systems using porous and disordered electrode surfaces, where diffusion effects depend on nanoscale topographical features (Lvovich 2012). Individual low frequency range measurement cycles take seconds-minutes to complete resulting in long cycle durations compared to high frequency tests. Increased low frequency measurement time can be reduced by combining frequencies into a multi-sine signal, but such an approach complicates analysis (Nam et al. 2013) and may increase vulnerability to electromagnetic interference (Albertini and Kleemann 1997). In contrast, identification and measurement of a small number of key frequencies where biomarker binding causes significant impedance changes can reduce measurement time without compromising functionality (Daniels and Pourmand 2007). Furthermore, including irrelevant frequency measurements can lead to errors such as artefacts from apparent inductive behavior (Anderson and Buhlmann 2016) or model fitting residuals, the deviation between data and regression model (Macdonald and Andreas 2014). Whilst this approach yields fewer data points for analysis, which is less desirable for model fitting algorithms since these rely on larger data sets to reduce fitting error (Carson et al. 2003), a more direct approach, such as comparison of voltage output to threshold values, can take advantage of pronounced impedance changes at specified response frequencies, and avoid the limitations of CNLS algorithms. Since conventional electrochemical equipment already processes all impedance data from a voltage signal, the effectiveness of this simplified method can be compared with existing methods.

**Table 1:** Developmental portable EIS devices in recent publication. Hz = hertz; PC = Personal Computer; μC = microcontroller; *Field-programmable gate array; †Electrocardiogram/Electromyogram

Group	Signal Source	Interface	Frequency range	Application
Conesa et al. 2017	FPGA*	PC	0.01 Hz to 10 MHz	Biofuel production
Chuang et al. 2016	AD5933	Custom	1 kHz to 100 kHz	Clinical biosensing
Ferreira et al. 2016	AD5933	Custom	5 kHz to 270 kHz	Body composition
Grassini et al. 2018	Arduino Due	Unspecified	0.01 Hz to 100 kHz	Corrosion testing
Harder et al. 2016	AD5933	Smartphone	3 kHz to 150 kHz	Body composition
Jiang et al. 2017	Smartphone	Smartphone	17 Hz to 17 kHz	Clinical biosensing
Lentka 2017	AD5933	PC	0.01 Hz to 100 kHz	Gas sensors
Liu et al. 2017	AD5933	Smartphone	100 Hz to 100 kHz	Clinical biosensing
Lu et al. 2015	Arduino Uno	PC	1 Hz to 2 kHz	Clinical biosensing
Piasecki et al. 2016	STM32F405RG μC	Custom	0.001 Hz to 100 kHz	Generic biosensing
Pruna et al. 2018	PIC24 μC	PC	0.1 Hz to 10 kHz	Clinical biosensing
Ravelomanantsoa et al. 2017	Unspecified μC	PC	512 Hz and 2048 Hz	Remote ECG/EMG†
Samanta and RoyChaudhuri 2016	ATMega8 μC	LCD screens	100 Hz to 100 kHz	Clinical biosensing
Tsai et al. 2014	AD5934	Custom	11 kHz to 191 kHz	Single cell analysis
Wen et al. 2017	PC	PC	60 Hz to 200 Hz	Food biosensing

Here we developed the POISED-5 instrument having sought to refine EIS instrument design to combine advantages of selecting a small number of discrete frequencies with a simplified impedance analysis, yet achieving resolution of the analog waveform matching current commercial FRA equipment. After demonstrating the effectiveness of the system by quantifying voltages that exceeded (low impedance) or undershot (high impedance) threshold values using solid state ‘standards’, the device was used to measure CA125, the gold standard biomarker for advanced stage ovarian cancer diagnosis and relapse following chemotherapeutic treatment (Razmi and Hasanzadeh 2018). Anti-CA125 antibodies were immobilized on a screen-printed graphene biosensor, and CA125 protein detected using the 5-frequencies EIS approach, demonstrating the potential for this system to be developed as a fully integrated point of care system.

## Materials and methods

### POISED-5 on-board EIS system design and evaluation

**Fig. 2:**
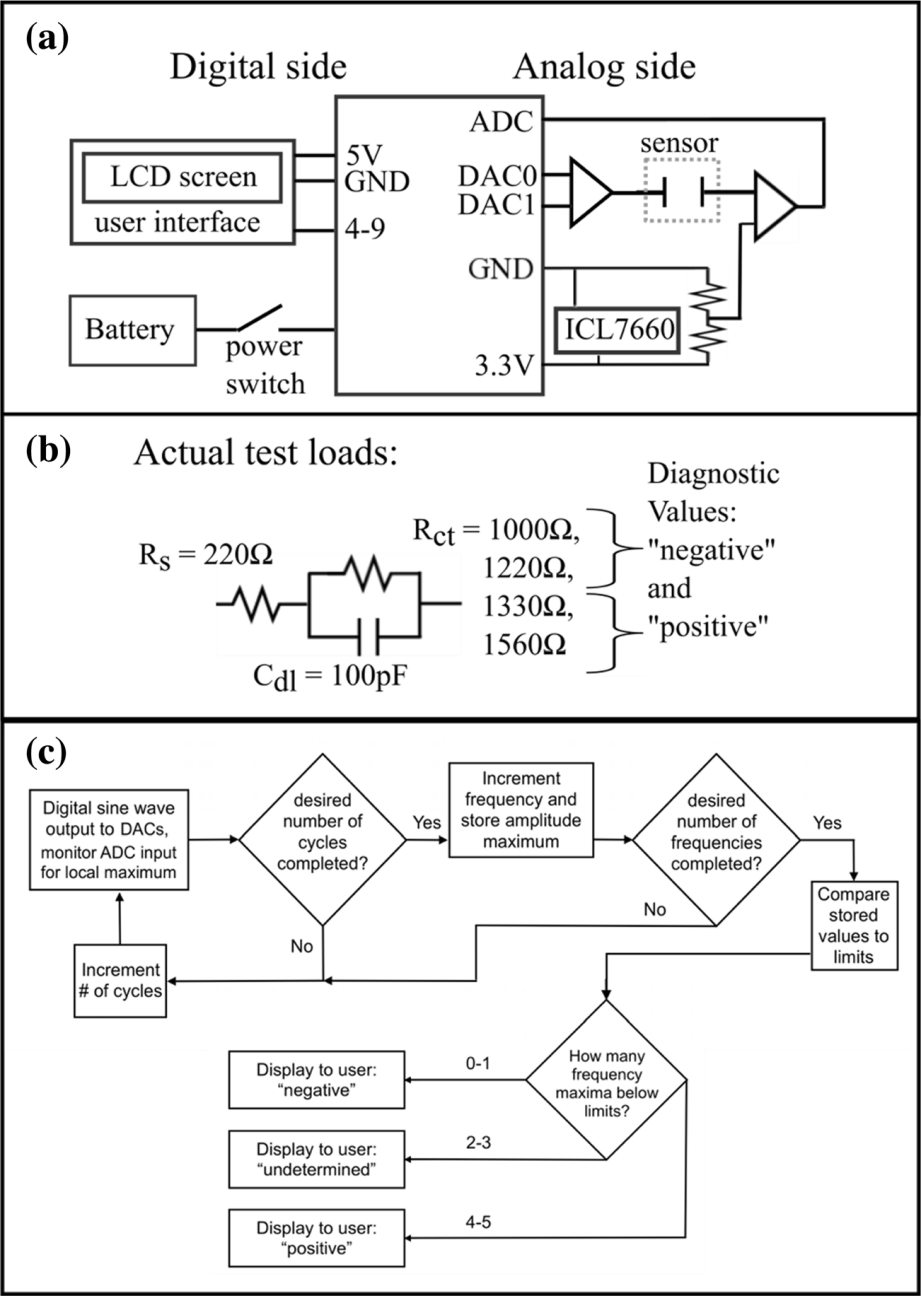
Device design. **a** Hardware diagram showing key inter-component connections. The Due’s digital output pins 4 through 9 provide control of the LCD screen; the 3.3 V output provides positive rail voltage to analog side components while the 5 V output energizes the digital user interface. An ICL7660 provides negative rail voltage to OpAmps. Some passive components have been excluded for clarity. **b** Solid-state test loads used to evaluate performance of the device and compare with the FRA equipment. **c** Concept of software applied by the device

The POISED-5 device was assembled by applying an Atmel SAM3X8E ARM Cortex-M3 controller on an Arduino Due board (Fig. 2a). The on-board Due 12-bit digital-to-analog converters (DACs) and 12-bit analog-to-digital converters (ADCs) provided outputs and inputs to the load through analog conditioning circuits. AD820 operational amplifiers (OpAmps) were used to perform analog functions, and an ICL7660 voltage converter provided a bipolar power source. The package was energized by either a rechargeable battery for portable functionality, or the Universal Serial Bus (USB) input that provides the ability to connect to an external processor to perform device maintenance. Input and output signal control plus all processing functions were performed via software stored on the on-board Due 512 kilobyte flash memory. The user interface was provided by a common commercial 16-pin liquid crystal display (LCD) screen. Electrochemical sensors connect to the POISED-5 through a cable connector (DRP-CAC, Metrohm).

Device performance was evaluated against a standard commercial electrochemical suite, Metrohm Autolab PGSTAT302N with FRA32M frequency response module, DRP-DSC boxed connector and Nova 2.0 interface software. The FRA is capable of multiple simultaneous sine waves, potential ranges up to 10 V and frequencies as low as 0.01 mHz reporting 0.003% resolution. Four test loads (Fig. 2b) were used to represent a hypothetical biosensor at various impedance values, and measurements at 28 mV_PP_, 0 V biased, and single sine waves at 0.67 Hz, 0.25 Hz, 0.15 Hz, 0.11 Hz and 0.087 Hz with two full periods at each frequency were compared. Signal generation was defined as a sine wave with amplitude of 0.01 V_RMS_, which corresponds to absolute amplitude of approximately 14 mV, or 28 mV from peak to peak (V_PP_). The five frequencies selected represent a span above and below 0.1 Hz, overlapping the range used in many sub-hertz impedance sensing studies. As in standard EIS, POISED-5 starts measurements at the highest frequency, continuing cycles until required response data has been stored in memory, then continuing to the next highest frequency. Output signal frequencies are progressively decreased by adding a fixed increment to the output voltage step delay. After completing cycles at 0.67 Hz each delay is increased by 7 milliseconds to give 11 s between steps, equaling 0.25 Hz. On the device any number of frequencies, cycles at each frequency and range of frequencies can be programmed by increasing loop counts or applying delays in units of microseconds. On the Metrohm FRA equipment, discrete frequencies and minimum cycle numbers were manually entered into the custom procedure on Nova software.

Due to the inherent interface differences between our device and the FRA equipment, interpretation of raw data was performed in two ways. Digital input maxima of the response signal were collected by the device and processed by customized code written in the Arduino integrated development environment (IDE) (Fig. 2c). In contrast, with the FRA impedance magnitude data were analyzed and collective impedance data was modelled to extract R_ct_ values using Nova software.

For the purpose of establishing performance, solid-state components were used to create test loads in the form of R(RC) circuits. These loads represent idealized systems rather than real sensor behavior to avoid variations from electrochemical sources. As many impedimetric biosensors report biomarker specific R_ct_ ranges test load values were selected to match typical R_s_ and R_ct_ values from an experimental impedimetric biosensor for CA125 measurement (Gazze et al. 2018). The test load C_dl_ value was limited by unpolarized capacitor size that remained constant for all loads since R_ct_ was assumed to have greater effect on impedance magnitude changes than C_dl_ in a faradaic sensor system. Hardware validation was performed with the lowest resistance (1000 Ω) test load. To assess the quality of generated and received waveforms, device signals on either side of the test load were captured on the IDE serial monitor while operating at the lowest measurement frequency (0.087 Hz).

Device precision was assessed by determining coefficient of variation (CV) of the maximum digital input value stored for each frequency before comparison with threshold values. In contrast to standard deviation, CV accounts for percentage of the mean making it the conventional metric for variability in clinical assays. Linearity and accuracy were determined by simulating a standard curve calibration with test loads of increasing R_ct_ value. Digital input maxima values stored by software were plotted and compared for each test load, and device reliability was determined based on the reproducibility of the diagnostic outcome for each test load. On the FRA, this assessment was based on the software’s declared |Z| values and fitted R_ct_ values. The validation test was repeated with ferrocyanide/ferricyanide electrolyte on unmodified screen-printed electrodes (SPEs) to quantify variability caused exclusively by electrochemical phenomena.

### CA125 measurements

To demonstrate compatibility of the proposed device with an existing experimental biosensor, measurements were performed on three CA125 biosensors functionalized according to the method detailed in Gazze et al. 2018. Briefly, the sensor is constructed by electropolymerizing aniline monomers (product #242284, Sigma Aldrich) dissolved at 0.1 M into a 1 M sulphuric acid solution (product code S/9240/PB17, Fisher Scientific) onto a commercial screen-printed graphene-modified carbon electrode (DRP-110GPH, Metrohm UK). After ten cycles between −0.1 V and 1.2 V against the SPE’s silver pseudo-reference electrode, active surfaces were rinsed with water (Millipore UK) and functionalized with antibody (MA5–12425, ThermoFisher Scientific) activated for surface immobilization with N-(3-Dimethylaminopropyl)-N′-ethylcarbodiimide hydrochloride (#E7750, Sigma Aldrich) and N-Hydroxysuccinimide (#130672, Sigma Aldrich). After each functional step, electrochemical characterization with cyclic voltammetry (one cycle from −0.7 to 0.7 V) and EIS (50 frequencies logarithmically distributed between 1000 and 0.05 Hz at 0.1 V bias, 10 mV amplitude) was performed in 5 mM K_n_[Fe(CN)_6_]^3−/4-^ (Sigma Aldrich) dissolved into PBS (P5368-10PAK, Sigma Life Science). Fluid samples (10 μL) of CA125 peptide (5609-MU, Bio-techne) in PBS were left on the sensor at room temperature for 15 min before rinsing with PBS, followed by application of 100 μL electrolyte to perform EIS for sample analysis.

To enable a more robust adaption of the CA125 sensor for 2-electrode operation, graphite (DRP-110, Metrohm UK) was used as the working electrode material, thereby matching the counter electrode. All other solutions and materials were prepared as above. Detection range and repeatability of sensor response was first assessed against graphene-based sensors by repeating calibration curve procedures on both electrode types for CA125 concentrations of 0.00096 ng/μL, 0.0048 ng/μL, 0.024 ng/μL & 0.12 ng/μL. Briefly, the lowest concentration was applied to the sensor for 15 min before rinsing and measurement followed by repeating the procedure with the next lowest concentration until impedance analysis was completed. These solutions were prepared from recombinant CA125 protein serially diluted into PBS.

## Results

### Device development

**Fig. 3:**
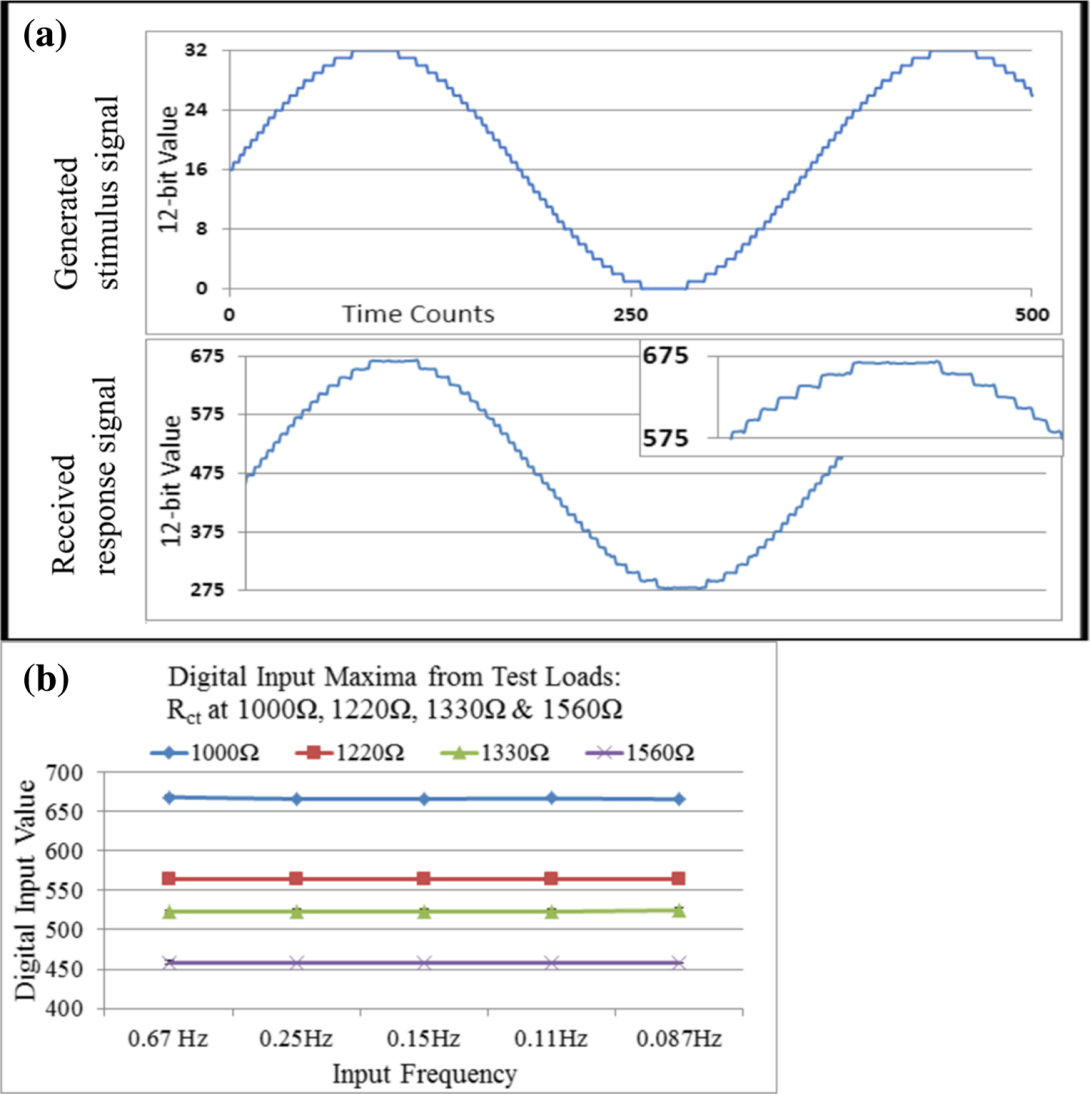
Device evaluation. **a** Waveform generated by device DAC, and received by device ADC, plotted from IDE serial monitor output. Units correspond to the controller’s 12-bit digital range values (0 to 1023). **b** Maximum digital values input by the device during triplicates performed on each test load, to simulate a sensor calibration curve. Black bars represent standard deviation

Initial investigations were conducted to verify the performance of the device (POISED-5) as a signal generator under ideal operational conditions. This validation was followed by performance testing on solid-state circuits and unmodified screen-printed electrodes. Device resolution was established by comparing output and input waveforms when connected to the 1000 Ω test load, and both device-generated and ADC-received sinusoids were symmetrical (Fig. 3a). Generated signals applied 32 distinct analogue values to achieve the desired milli-volt output range, due to the maximum available 12-bit range (1024 values) spanning 0.5 to 2.75 V. Input waveforms conditioned by the OpAmp circuit spanned approximately 400 distinct values, corresponding to a range of 0.7 to 1.8 V. Some distortion was apparent on the received waveform, but noise was not seen to compromise the stability of crest values.

Following hardware validation, each of four test loads was connected to the device and measurements taken at 5-frequencies to determine input signal ranges for device calibration. Current out of the load was amplified and converted to voltage by the OpAmp circuit, then received at the ADC as a digitalized value from 0 to 1023. Higher currents resulted in higher input values, which indicated proportionally lower load resistances. Maximum digital input values corresponding to response currents were inversely proportional to load impedance (Fig. 3a). Precision was sufficient to distinguish each load by its input maxima with the highest CV equal to 0.35% of the mean within same-frequency repeats on the same load. By defining the higher load resistances (1330 Ω and 1560 Ω) as standards for positive diagnostic indication, code was calibrated to display the word “positive” when maximum input values at each frequency remained at or below those from the 1330 Ω load. Lower-resistance loads yielded higher input values, which were programmed to display “negative” when consistently above the assigned threshold. A confidence level system was implemented to group results by response ranges. Four or five input maxima below threshold was deemed positive, zero or one was deemed negative, and two or three maxima under threshold values was classed as “undetermined”. Any measurements producing an “undetermined” result would indicate either inappropriate choice of threshold value or inadequate reliability between repeats. In subsequent tests, measurements gave “positive” results when actual test load resistance was equal or above 1330 Ω, and “negative” when load resistance was below 1330 Ω, no measurements produced an “undetermined” result. The POISED-5 device was able to quantify the impedance of solid-state test loads from received digital input values. Device software was calibrated with threshold impedance based on observed digital input value maxima at each frequency, which were repeated with high precision between measurements.

**Fig. 4:**
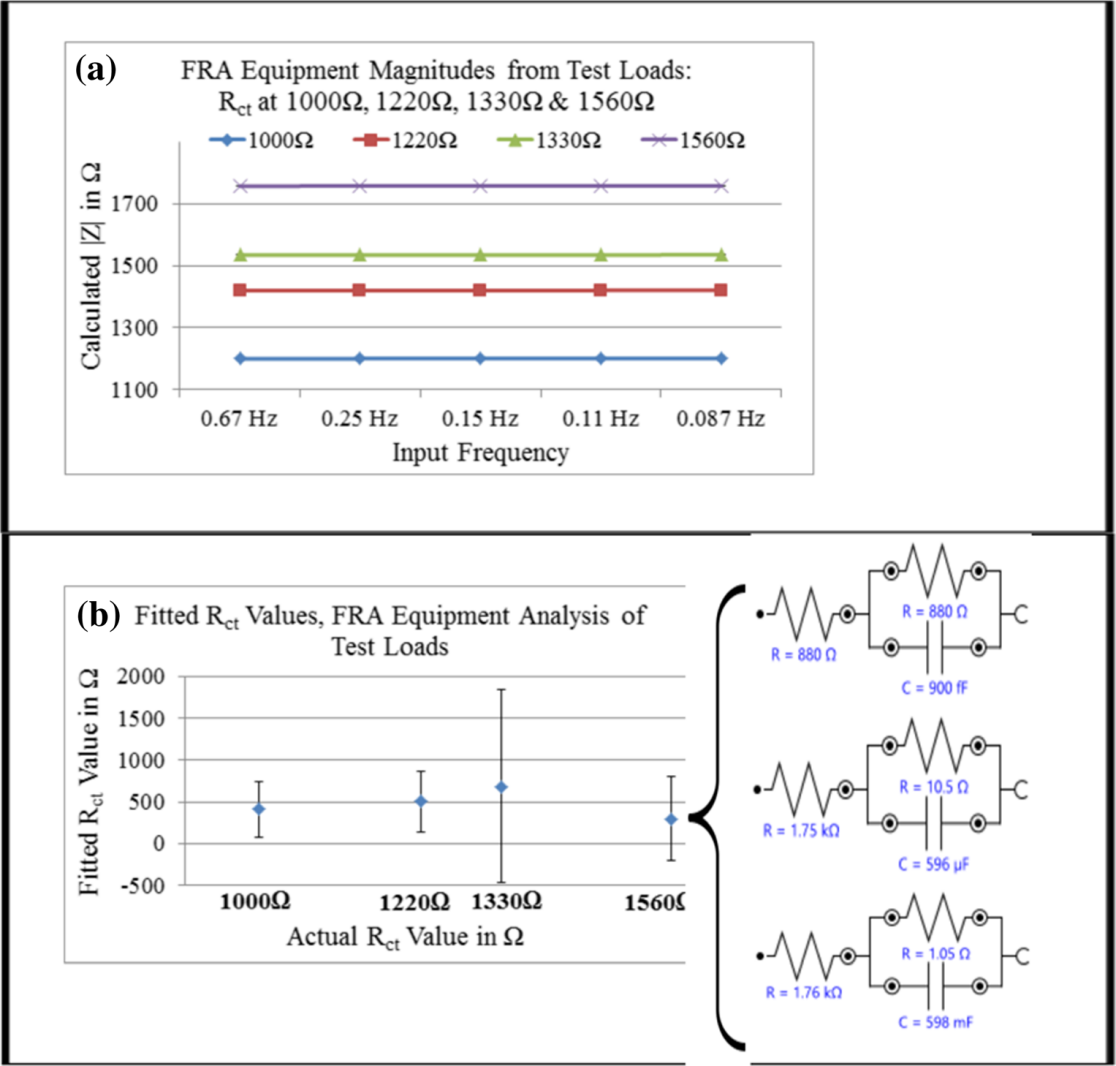
Comparison between the device and commercial electrochemical equipment (**a**) calculated |Z| values for FRA-received responses during triplicates performed on each of 4 test loads, to simulate a sensor calibration curve. Black bars represent standard deviation. **b** R_ct_ values calculated for the test loads using Nova software’s proprietary fitting algorithm, with bars representing standard deviation. The fitted models from Nova software for the 1560 Ω test load repeats are depicted as exported

Comparison of POISED-5 and Metrohm FRA performance following identical EIS measurement was made using four test loads, operating in two-electrode setup. Output voltage vs. time and input current vs. time (Nova software, data not shown) confirmed the receipt of a low-noise signal from the test load. System generated response data including calculated model components and magnitude values were exported from Nova for statistical analysis. Impedance magnitude (Fig. 4a) showed high precision between repeats on the same load, with CV values for repeats ranging from 0.099% to 0.00729% of the mean. Despite this, fitting the impedance data to the equivalent circuit model (Fig. 4b) did not approximate the resistor values in the test loads. The R_ct_ values calculated for test loads were not significantly different, and coefficients of variation ranged from 72.3% of the mean (1200 Ω load) to 169.8% of the mean (1560 Ω load). Each test load could be distinguished from Nova calculated magnitude values with very high precision, without requirement for model fitting. Impedance magnitudes were also similar between frequencies for each solid-state test load, but Nova analysis software did not provide a tool to simultaneously compare with a standard value to determine diagnostic significance. When fitting analysis was applied to match the load to its real-world model, none of the load resistors could be identified by Nova software with accuracy or reliability, even in the absence of electrochemical variables.

**Fig. 5:**
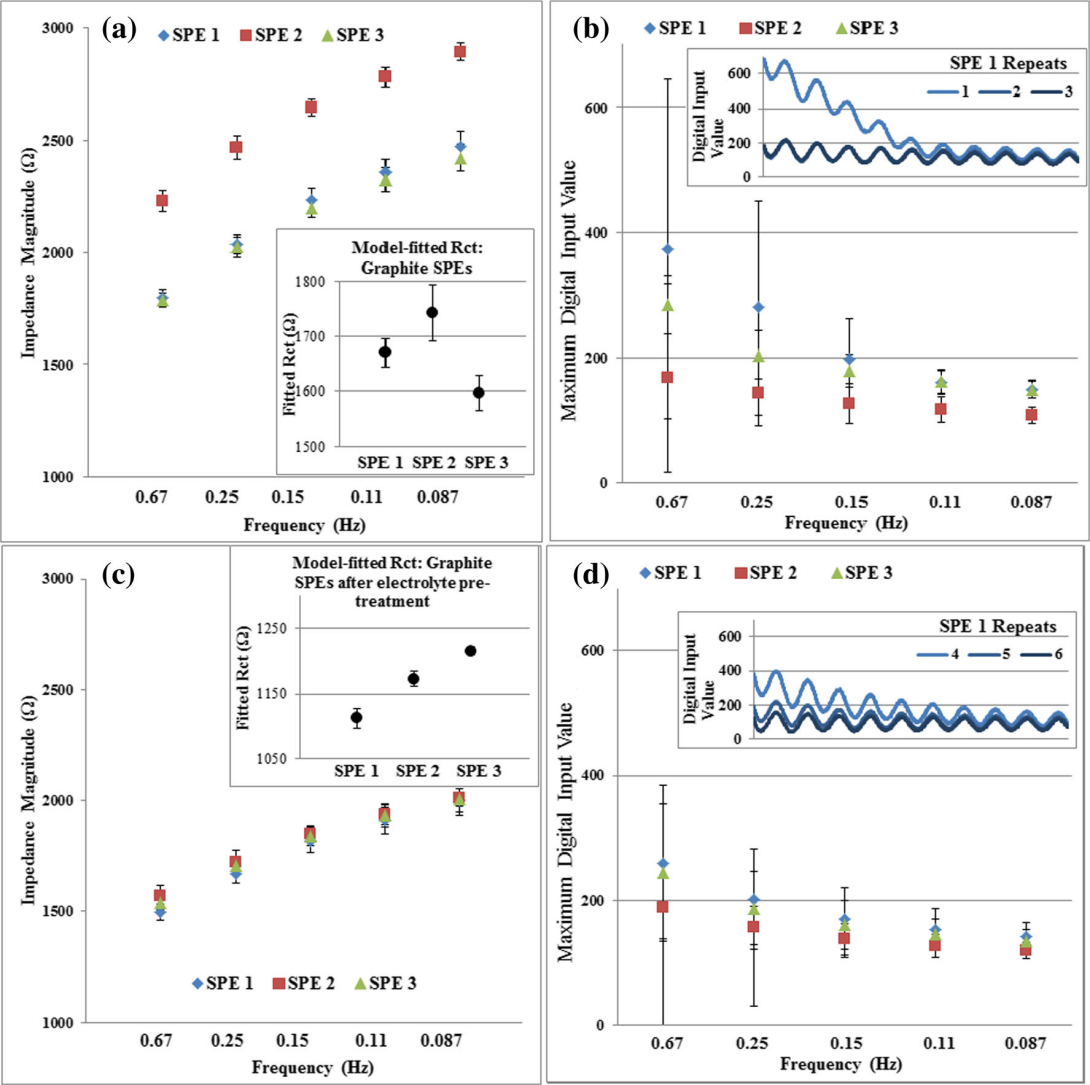
Impedance precision of unfunctionalized graphite electrodes, using the proposed 5-frequency EIS on (**a**) Metrohm FRA equipment and (**b**) the POISED-5 device. Inset to (**b**) shows actual response inputs to the device ADC over the measurement duration for SPE 1. After pre-treating the same electrodes with electrolyte for 10 min prior to commencing measurement sets, the precision test was repeated with (**c**) Metrohm FRA and (**d**) POISED-5. Inset to (**d**) shows actual response inputs to the ADC for SPE 1

POISED-5 was evaluated for use with an electrochemical system through a standard electrolyte on graphite electrodes. Applying the 5-frequency measurement in a 2-electrode setup, the impedance of three unmodified graphite SPEs was assessed and compared to the Metrohm FRA equipment. As in solid-state load tests, FRA-measured impedances were interpreted with magnitude and fitted model components calculated by Nova software, while POISED-5 performance was assessed by recording input values received at the ADC. With the Metrohm FRA, precision of magnitude and fitted resistance was high between repeats performed, and differences between SPEs were pronounced (Fig. 5a). When measured using POISED-5, SPE impedances increased on each sequential repeat with the greatest changes occurring during the beginning of the first measurement, suggesting a degree of temporal instability is associated with the graphite/electrolyte system (Fig. 5b). When the electrode surface was pre-treated with electrolyte for 10 min prior to measurements, standard deviation decreased using both FRA and POISED-5 (Fig. 5c, d), with the latter showing improved stability between repeats with each load (inset to 5d). Despite indications of instability from electrochemical sources, the POISED-5 device was able to quantify impedance of SPEs based on input values stored during measurements. Comparing mean maximum input values to tests on solid-state loads, responses from graphite SPEs indicated charge-transfer resistances higher than 1560 Ω. Impedance markers on the same SPEs measured with the Metrohm FRA equipment gave greater inter-repeat precision, but analysis with magnitude and fitted resistance amplified differences between individual SPEs.

### Detection of ovarian cancer biomarker CA-125

Previously we have demonstrated the utility of screen-printed graphene biosensors as a label-free platform for the detection of CA125 using EIS measurements on the Metrohm FRA platform (Gazze et al. 2018). The sensor was fabricated through deposition of a polyaniline layer via electropolymerization on to a graphene screen-printed electrode functionalized with anti- CA125 antibody via covalent cross linking to polyaniline. Using this system CA125 measurements were made across a dynamic range of 0.92 pg/μL – 15.20 ng/μL. Here, having established the theoretical concept of limited-frequency data collection and on-board analysis, we sought to evaluate the ability of system to directly measure CA125 biomarker, and compare to our previous report.

**Fig. 6:**
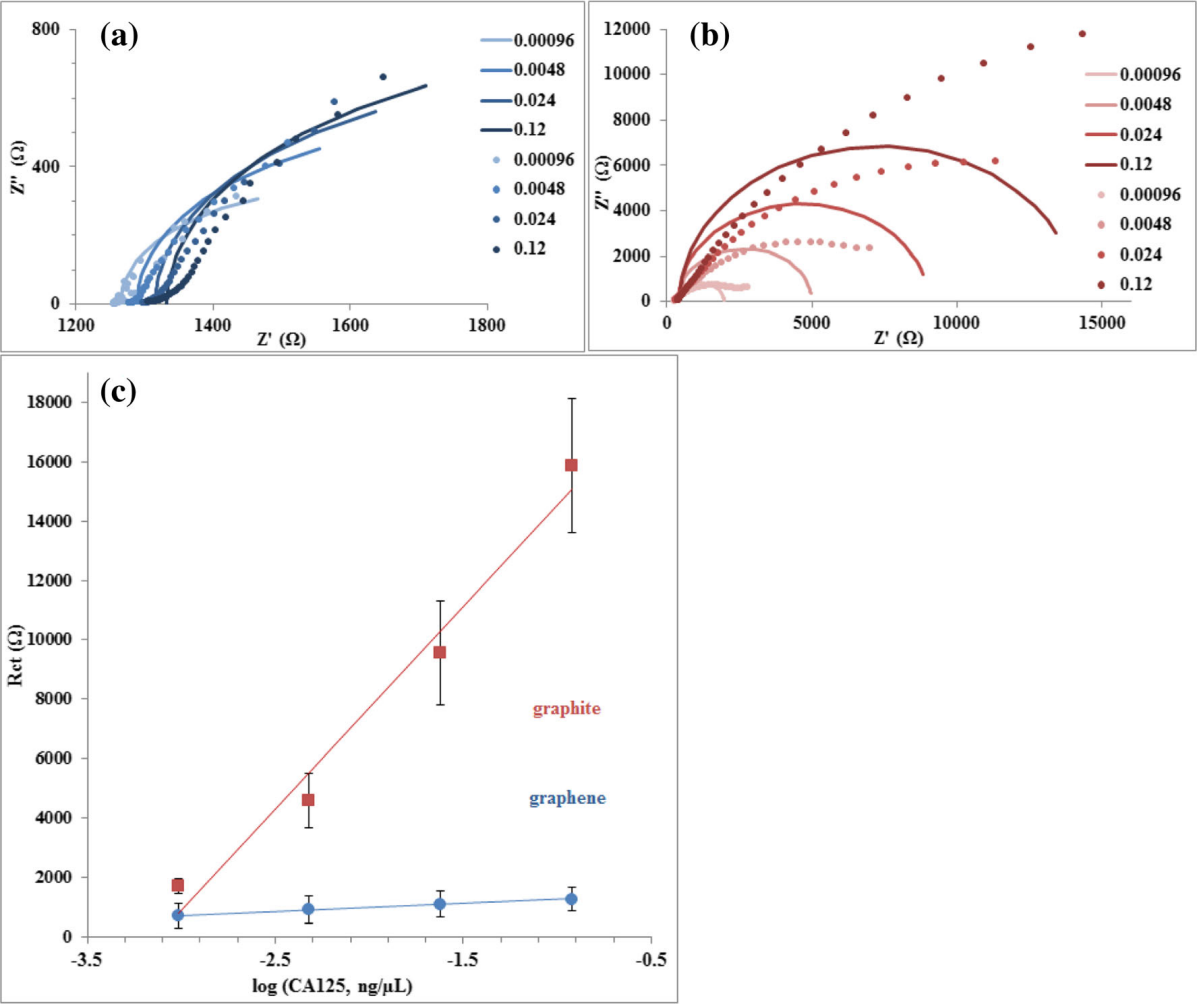
Nyquist plots show actual (dot) and model-fitted (line) impedance response to CA125 protein (in ng/μL), on anti-CA125 antibody functionalized (**a**) graphene electrodes and (**b**) graphite electrodes. Standard curves (**c**) show mean R_ct_ vs. logarithm of CA125 concentration on each material; bars represent standard deviation

To establish equivalency of the graphite-based sensor to graphene sensors, an established calibration procedure (Gazze et al. 2018) was performed on anti-CA125 antibody functionalized graphene and graphite SPEs. Sensor impedance was determined by fitting to an R(RC) equivalent circuit using Nova analysis software. With both graphite and graphene sensors, Nyquist plots confirm progressive increases of Z’ and Z” with each increase in CA125 peptide concentration, most apparent from changes at lower frequencies (Fig. 6a & b). Sensors functionalized on graphite electrodes respond to each CA125 concentration with higher real and imaginary impedances than graphene electrodes, as expected due to lower conductivity of the more disordered carbon material. Model-fitted impedances of graphite electrodes corresponded to higher charge-transfer resistances, which increased after CA125 exposure with a steeper gradient than the graphene-based sensor (Fig. 6c). Despite higher inter-repeat variation, graphite-based sensors were more responsive to CA125 than graphene-based sensors at the levels tested, reducing ambiguity of sensor response between concentrations.

Having established that graphite sensors give enhanced performance for CA125 detection using a 3-electrode system we sought to simplify the biosensor by using 2-electrode measurements to permit compatibility with the POISED-5 and to avoid variables caused by different redox reactions on the graphite/graphene and silver reference electrodes. Maximum input values from responses to each CA125 concentration measurement were analysed to establish a standard curve. Maximum values recorded in measurements on the first two sensors were used to calibrate the user readout to display “positive” or “negative” after measurement on a third sensor. Applying a threshold value for each frequency based on observed responses permitted performance testing for diagnostic determination.

**Fig. 7:**
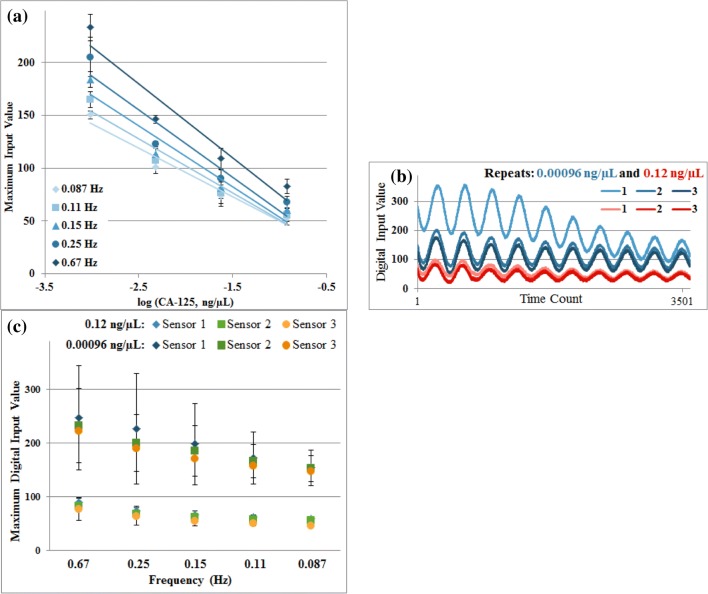
Device-measured sensor impedance after 15-min exposures to CA-125 in PBS, (**a**) standard curve of maximum input values at each frequency vs. log of concentration; (**b**) stability of input values for Sensor 1 after exposure to CA125 at starting and ending concentrations; and (**c**) maximum input values for each frequency at starting and ending concentrations; bars represent standard deviation

Comparing maximum input values received by the device for each frequency as the concentration of CA125 increased confirmed the inverse relationship between voltage received by the device and sensor impedance (Fig. 7a). Small deviations of maximum input values recorded for each concentration demonstrate high precision of quantification between individual sensors, giving distinct signal levels for each sample. Calculated precision of input values recorded by POISED-5 at 0.087 Hz ranged from a CV of 0.6176% to 4.562% of the mean. This exceeded the detection precision of the 3-electrode 50-frequency Metrohm FRA measurement, which gave CV from 14.193% to 19.943%.

Decreasing input values between repeated measurements revealed some instability on each sensor, suggesting increased impedance with each subsequent measurement (Fig. 7b). However, the effect of this instability between repeats had less impact on later measurements with higher concentrations of CA125 (Fig. 7c). The final sensor was tested with the POISED-5 programmed to display “positive” for impedances above 0.024 ng/μL by comparing response inputs at each frequency to assigned threshold values. In measurements after exposure to CA125 concentrations at or below 0.024 ng/μL results were classified as “negative”, and “positive” with the highest CA125 concentration. For the measurement parameters selected, the POISED-5 device was able to detect impedance increases of the CA125 sensor through response voltage at each applied frequency and demonstrates the potential for determining threshold impedance levels for diagnostic use.

## Discussion

Here we have developed a novel system, POISED-5, which has addressed several requirements essential for portable biosensor devices, including removing the need for connection to an external processor achieved by identifying, and implementing fewer frequency numbers for optimized biomarker detection. The POISED-5 concept is aimed at achieving the simplest possible EIS measurement device for a specific purpose based on the requirements for accurate determination of a specific clinical biomarker, measured at the POC.

To achieve this, we demonstrated that the Arduino Due SAM3X controller can provide more than adequate signal resolution, memory storage space and processing speed. At a clock rate of 84 MHz, the controller can generate DAC output cycles below 1 Hz without risk of slowing other processes, nor interference from AC power sources. Whilst other Arduino boards produce only pulse-width-modulated (PWM) signals, which default to a frequency ~490 Hz, the Due features a two-channel DAC, giving a true analog output value. Processor speed manipulation could reduce the frequency generated by PWM outputs, but this would require setting multiple timer speeds to produce more than one measurement frequency, slowing down other functions. Also, the square wave generated by PWM requires additional analog signal conditioning to produce an equivalent sine signal, a complexity that was unnecessary when using a DAC-equipped controller. Maximum response signal amplitudes sampled for each frequency are stored then compared to values with diagnostic significance after the measurement is completed, a function that occurs instantaneously due to the short processing time of our direct approach to impedance interpretation. By assuming biosensor impedance magnitude increases proportionally with increased biomarker capture, complex plotting and modelling was replaced with direct comparison to threshold input voltage values.

Five frequencies in the sub-hertz region were selected for their ease of implementation in direct impedance analysis. Validation on solid-state test loads confirmed a precise response voltage was received in proportion to load resistance at each frequency applied. Whilst commercial FRA equipment can provide precision impedance data for the low-voltage, low-frequency measurements, it does not provide a useful modelled representation of the response signal when only 5 frequency points are used. In contrast POISED-5 was able to quantitatively distinguish each test load value with precision comparable to the FRA equipment’s |Z| determination, demonstrating that response signal impedance can be interpreted without calculation of complex components or model fitting. POISED-5 2-electrode measurements indicated precision at low frequencies improved with repeated applications as instability was observed in measurements obtained following preliminary signal application. Whilst this instability was not observed on the FRA-equipped potentiostat, the equipment gave a different impedance magnitude and charge-transfer resistance for each electrode tested. Exposing electrodes to electrolyte in advance did not completely remove the inter-electrode differences in calculated R_ct_, while POISED-5 precision of input signals at the lowest applied frequency reliably quantified impedances without any inter-electrode differences.

POISED-5 could be readily adapted to measure inputs from a range of impedimetric biosensors as the measurement parameters can be customized through minor software adjustments or hardware component additions. Adding bias voltage through software is also possible as the output signal uses less than 5% of the available DAC range. However, as the analog circuitry is optimized for the existing DAC range, installation of a summing OpAmp to add the desired bias would offer a more robust solution. A fixed value up to 3 V could be added using a voltage divider in the same way a bias was added to the response signal before input to ADC in the reported device. Accommodating a capacitive biosensor would likely require addition of a phase change detection capability, which could also be implemented by adding an OpAmp circuit for comparison of two sine wave signals. Although this would produce another arbitrary digital value rather than calculating the actual phase change, we have already demonstrated that translating proportional values into effective impedance changes can be used for a semi-quantitative diagnostic purpose. Finally, two OpAmps are needed to condition analog signals between the Due and the biosensor, therefore multiplexing for simultaneous measurement on more than one biosensor would require replication of these components and connecting sensor response outputs to other ADC inputs available on the existing controller. The 12 separate analog inputs on the Due could theoretically allow expansion of the device to accommodate up to a dozen sensors.

Other approaches to portable EIS include using the commercial Analog Devices (AD) integrated circuit (IC) impedance analyzer AD5933 (Ghafar-Zadeh et al. 2010). This chip combines sine wave generation, ADC, basic impedance analysis and allows multi-sine functions with discrete Fourier transform processing. However, its range of waveform parameters does not include low-amplitude or low-frequency EIS measurements (Hoja and Lentka 2010). To convert output signals from the AD5933 into low-voltage low-frequency ranges required for biomarker detection, additional analog components need to be connected, adding complexity and cost (Al-Ali et al. 2018; Margo et al. 2013).

Several attempts have been made to develop cell phones as a user interface for point-of-care devices (Ainla et al. 2018; Dou et al. 2016; Jiang et al. 2014; Salomon et al. 2014). A separate piece of hardware couples to the sensor while the smart phone controls measurement parameters and displays results through custom applications. In some cases, the phone platform is also used as an EIS signal generator and an impedance analyzer (Zhang et al. 2015). While smart phones are considered ubiquitous even in non-traditional health care settings, regulatory barriers prevent using a personal communication device as a quality-controlled diagnostic tool (Kwon et al. 2016).

Most experimental SPE-based biosensors are designed for measurement with potentiostats on 3-electrode systems, which complicates hardware implementation and increases equipment size and cost. A dedicated reference electrode theoretically improves measurement stability, such as in determining open circuit potential (OCP). To account for material and surface area differences between electrodes, EIS is considered most reliable at a bias voltage equal to the OCP (Bard and Faulkner 2001). Detection of OCP requires a separate operation to detect the millivolt-range potential between electrodes caused by redox reactions between electrodes and electrolyte. An OCP bias feature might favor existing experimental biosensor designs, but a more practical solution would be to adapt sensors for 2-electrode measurement. To illustrate this principle, and to demonstrate the utility of POISED-5, our previously reported experimental impedimetric biosensor for the ovarian cancer biomarker CA125 was redesigned as a 2-electrode format. The graphene electrode material was replaced with graphite without any performance losses; in fact the graphite SPE system delivered clearer concentration-dependent impedance measurements. These changes were detected during the 90-s 5 frequency measurement, allowing faster determination of CA125 than with a 50-frequency measurement on the FRA system and equivalent circuit modelling.

The current use of CA125 measurement is in the diagnosis and monitoring of ovarian cancer and is based on measurement of a concentration threshold in serum, where levels over 30–35 U/mL are considered indicative of disease, while lower concentrations are considered in the normal range (Felder et al. 2014). Based on the sensitivity achieved with a 2-electrode graphite SPE system, POISED-5 offers a POC system that could be used for initial CA125 screening in a community setting (family doctor or pharmacy). The device demonstrated reliability as a handheld platform for portable biosensing, employing a shortened frequency range and simplified impedance processing for fast EIS measurements, and offers the potential to deliver a new paradigm for impedimetric biosensors, enabling effective deployment of sample testing systems.
